# Electrochemical Detection of Melphalan in Biological Fluids Using a g-C_3_N_4_@ND-COOH@MoSe_2_ Modified Electrode Complemented by Molecular Docking Studies with Cellular Tumor Antigen P53

**DOI:** 10.1021/acsomega.4c00558

**Published:** 2024-04-30

**Authors:** Nevin Erk, Gülbin Kurtay, Wiem Bouali, Zeyneb Gülsüm Sakal, Asena Ayşe Genç, Zeliha Erbaş, Mustafa Soylak

**Affiliations:** † Ankara University, Faculty of Pharmacy, Department of Analytical Chemistry, 06560 Ankara, Turkey; ‡ Hacettepe University, Faculty of Sciences, Department of Chemistry, 06800 Ankara, Turkey; § Ankara University, Graduate School of Health Sciences, 06110 Ankara, Turkey; ∥ Yozgat Bozok University, Science and Technology Application and Research Center, 66200 Yozgat, Turkey; ⊥ Erciyes University, Technology Research & Application Center (TAUM), 38039 Kayseri, Turkey; 6 Turkish Academy of Sciences (TUBA), Çankaya, Ankara 06670, Turkey

## Abstract

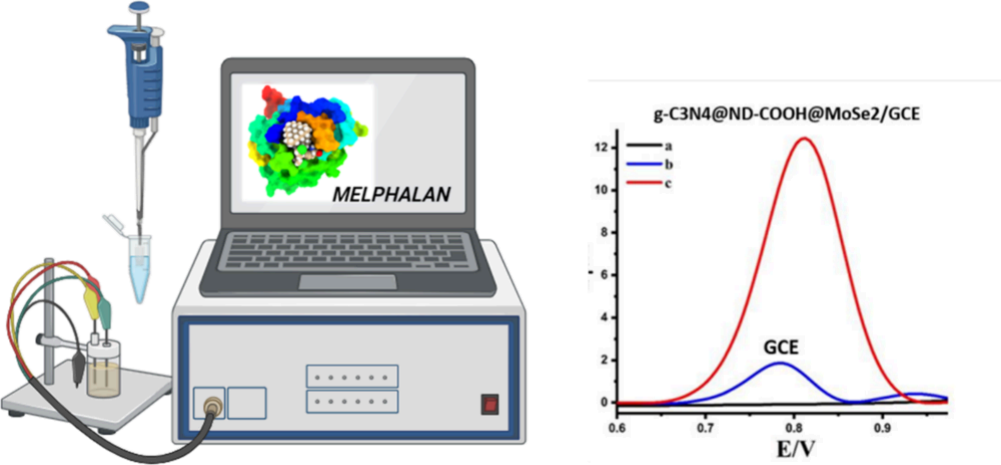

Melphalan (Mel) is a potent alkylating agent utilized in chemotherapy treatments for a diverse range of malignancies. The need for its accurate and timely detection in pharmaceutical preparations and biological samples is paramount to ensure optimized therapeutic efficacy and to monitor treatment progression. To address this critical need, our study introduced a cutting-edge electrochemical sensor. This device boasts a uniquely modified electrode crafted from graphitic carbon nitride (g-C_3_N_4_), decorated with activated nanodiamonds (ND-COOH) and molybdenum diselenide (MoSe_2_), and specifically designed to detect Mel with unparalleled precision. Our rigorous testing employed advanced techniques such as cyclic voltammetry and differential pulse voltammetry. The outcomes were promising; the sensor consistently exhibited a linear response in the range of 0.5 to 12.5 μM. Even more impressively, the detection threshold was as low as 0.03 μM, highlighting its sensitivity. To further enhance our understanding of Mel’s biological interactions, we turned to molecular docking studies. These studies primarily focused on Mel’s interaction dynamics with the cellular tumor antigen P53, revealing a binding affinity of −5.0 kcal/mol. A fascinating observation was made when Mel was covalently conjugated with nanodiamond-COOH (ND-COOH). This conjugation resulted in a binding affinity that surged to −10.9 kcal/mol, clearly underscoring our sensor’s superior detection capabilities. This observation also reinforced the wisdom behind incorporating ND-COOH in our electrode design. In conclusion, our sensor not only stands out in terms of sensitivity but also excels in selectivity and accuracy. By bridging electrochemical sensing with computational insights, our study illuminates Mel’s intricate behavior, driving advancements in sensor technology and potentially revolutionizing cancer therapeutic strategies.

## 1 Introduction

**Figure 1:**
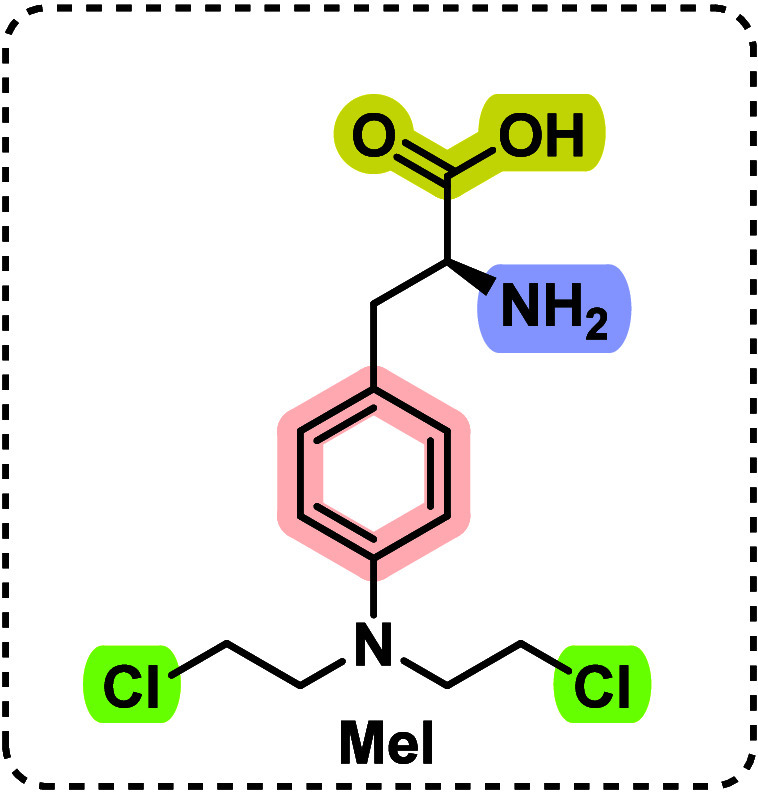
Chemical structure of melphalan (Mel).

Melphalan (Mel) [(2S)-2-amino-3-{4-[bis(2-chloroethyl)amino]phenyl}propanoic acid], commercialized under the brand Alkeran (Figure 1), stands as a potent anticancer pharmaceutical deployed against a variety of malignancies including multiple myeloma, breast cancer, advanced ovarian cancer, childhood neuroblastoma, and polycythemia vera.^1^

Functioning as an alkylating agent, Mel impedes DNA replication through forming covalent bonds with nucleophilic atoms in biological entities, thereby damaging DNA and obstructing its self-replication capability. By cross-linking atoms of DNA strands, Mel further inhibits DNA transcription.^2,3^ Despite its therapeutic promise, Mel administration is associated with a spectrum of side effects such as bone marrow depression, nausea, hair loss, fatigue, diarrhea, and rash, underscoring the necessity for precise, rapid, and cost-effective methodologies for Mel quantification.^4^

Historically, the detection of Mel has been pursued through chromatographic methods, paper-based electrochemical aptasensors, and differential pulse voltammetry, albeit with inherent limitations including high costs, time-intensive procedures, complex equipment requisites, and elevated detection limits.^5−9^ The quest for simpler, cost-efficient, and user-friendly analytical tools endowed with heightened sensitivity, selectivity, and swiftness necessitates the exploration of novel methodologies to achieve low detection limits for Mel quantification. In this milieu, electrochemical sensors emerge as viable candidates for clinical chemistry applications, offering attributes of high sensitivity and selectivity, rapid response, and economic feasibility.^10^ Several voltammetric sensors, such as cyclic voltammetry (CV), differential pulse voltammetry (DPV), square wave voltammetry (SWV), and electrochemical impedance spectroscopy (EIS), have introduced a new era of straightforward, cost-effective, rapid, and highly sensitive techniques for both qualitative and quantitative analyses in the fields of food and pharmaceuticals.

In recent years, notable advancements have been achieved in the development of electrochemical sensors leveraging nanomaterials and polymers. These sensors exhibit improved sensitivity, selectivity, and detection limits across a broad spectrum of analytes.^11,12^

The effectiveness of electrochemical sensors can be greatly enhanced by modifying electrode surfaces.^13^ Various types of composites, including carbon-based, metal-based, and polymer-based composites, have been developed to modify electrodes. Carbon-based composites, such as carbon nanotubes and graphene, have been acknowledged for their capacity to improve the sensitivity and stability of electrodes.^14^

In recent times, g-C_3_N_4_ has garnered considerable scientific interest due to its distinctive structure, defined by a π-conjugated polymeric network mostly composed of covalently linked carbon and nitrogen atoms. This material, which possesses a unique structure, is widely recognized as the most stable variant of carbon nitride. It exhibits numerous notable characteristics, such as its semiconducting properties, two-dimensional layered arrangement, lack of metallic elements, straightforward manufacturing process, compatibility with biological systems, and nonhazardous nature. The electrical structure of this material is highly flexible, and it possesses reactive spots on its surface, resulting in a significant surface area that facilitates electron donation and acceptance. As a result, it has found various uses in domains such as sensing and catalysis.^15,16^

Moreover, the integration of g-C_3_N_4_ with other substances has been employed to produce electrochemical sensors that exhibit enhanced selectivity and sensitivity. An example of enhanced electrocatalytic activity in diverse processes, such as oxygen reduction, hydrogen evolution, and carbon dioxide reduction, was seen by integrating g-C_3_N_4_ with metal-based materials. Incorporating g-C_3_N_4_ into carbon-based materials, such as graphene and carbon nanotubes, has been demonstrated to augment the electrochemical properties of these materials. These advancements encompass enhancements in electrical conductivity and an augmentation in surface area, resulting in an improved electrochemical efficacy. An enhancement in the sensing efficacy of estradiol sensors has been observed through the incorporation of g-C_3_N_4_ with metal-based nanoparticles. This improvement is attributed to the enhanced electron transfer kinetics and increased specific surface area.^17^

In recent decades, there has been considerable interest in carbon-based materials due to their notable thermal stability and mechanical qualities. Carbon nanomaterials, including nanodiamonds, fullerenes, and graphene, have found applications not only in drug delivery but also in cancer imaging and other medical fields^18^ Diamond, a metastable allotrope of carbon,^19^ stands out as one of the most prominent carbon compounds. Diamonds are extraordinary substances characterized by their unique physical and chemical attributes. The material exhibits notable hardness, exceptional thermal conductivity, and a high degree of chemical stability. The luminescence displayed by nitrogen-vacancy color centers in diamonds is highly intense, and the substance itself possesses nontoxic and biocompatible properties.^20^ Nanodiamonds have a notable chemical characteristic that renders them well-suited for utilization in medical contexts, namely, within medication delivery. The feature in question is ascribed to the surface’s inherent capacity to undergo facile modifications with additional molecules or functional groups.^21^ Moreover, the inclusion of diverse functional moieties, encompassing delocalized π-electron systems and oxygen-containing groups, on the nanodiamond (ND) surface enhances its electrical conductivity. Consequently, integrating ND into electrochemical analyses augments the electrocatalytic capabilities of the sensing apparatus, thereby enhancing its analytical efficacy.^22^

One strategy for improving the interaction between pharmaceuticals and nanodiamond surfaces is the alteration of the functional groups that are already present on the surface of the nanodiamond. Various functional groups, such as amine, amide, alcohol, carbonyl, and carboxyl, were examined for this particular objective. The carboxylic acid functional group has been suggested as a viable mediator for facilitating the interaction between nanodiamond surfaces and pharmaceutical compounds within functional groups. The enhancement of nanodiamonds’ efficacy in drug delivery applications can be achieved through surface modification, namely, by substituting pre-existing functional groups with carboxylic functional groups.^23,24^

The unique electric, optical, and mechanical characteristics of molybdenum diselenide (MoSe_2_), a material with a two-dimensional structure, have garnered significant attention within the materials science community.^25^ This phenomenon has garnered considerable interest across multiple domains of scientific inquiry, including but not limited to energy storage, catalysis, supercapacitors, and sensing.^26^ Extensive efforts have been dedicated to leveraging MoSe_2_ nanostructures to create exceptional sensing platforms through conductivity enhancement and surface modification for electrocatalytic oxidation processes. The distinctive 2D structures and heightened surface activities of MoSe_2_ nanosurfaces provide active sites conducive to target adsorption and catalytic redox processes within the sensing platforms.^27^ The integration of MoSe_2_ with carbon-based materials has been found to boost electrical conductivity, leading to an improvement in the electrochemical performance of the composite material.^28^

The concurrent utilization of MoSe_2_, ND-COOH, and g-C_3_N_4_ nanomaterials has demonstrated a synergistic phenomenon, leading to the enhancement of both the physical and chemical properties. In the present study, a facile approach was adopted to detect the alkylating agent Mel using a glassy carbon electrode (GCE) sensor modified with g-C_3_N_4_@ND-COOH@MoSe_2_. The sensor’s performance was meticulously evaluated on actual samples to assess the sensitivity and selectivity of Mel detection. Through optimization of parameters such as electrolyte solution, composite concentration for electrode modification, and electrolyte pH, the developed electrode exhibited optimum performance.

In the endeavor to derive a deeper molecular understanding of the interactions implicated in the electrochemical detection of Mel, molecular docking studies were conducted. Molecular docking serves as a potent tool to envisage the probable orientations and binding affinities when two molecules interact in a complex, thereby providing a molecular-level elucidation of the interactions. The choice of the cellular tumor antigen P53 (p53) for molecular docking with Mel is rooted in its paramount biological significance. p53, often dubbed as the “guardian of the genome,″ is a pivotal tumor suppressor protein known for its role in regulating cell cycle, apoptosis, and DNA repair mechanisms^29^ The interaction between chemotherapeutic agents like Mel and crucial cellular entities like p53 could potentially unveil molecular dynamics that may impact therapeutic outcomes. Furthermore, understanding such interactions can offer novel insights into the mechanistic actions of Mel, which is integral for advancing therapeutic strategies and drug design. Additionally, to better elucidate the rationale behind employing nanodiamond-COOH (ND-COOH) in sensor design, molecular docking studies were extended to explore the interaction between ND-COOH and Mel. The carboxyl functional group on the surface of ND-COOH can act as a bridge, facilitating the interaction between the nanodiamond surface and Mel. This pioneering endeavor utilizing an electrochemical sensor for Mel detection unveils a promising avenue employing g-C_3_N_4_@ND-COOH@MoSe_2_ as a potent tool for monitoring Mel concentrations in drug formulations and biological specimens.

## 2 Experimental Section

### 2.1 Material and Instrumentation

Sigma-Aldrich Co. (https://www.sigma-aldrich.com, Germany) provided ascorbic acid, d-glucose (99.5%), dopamine, potassium nitrate, l-arginine (98.0%), l-methionine, sodium sulfate, uric acid (99.0%), and potassium hexacyanoferrate (III) (K_3_Fe (CN)_6_, 99.5%). As the supporting electrolyte, acetic acid, boric acid, potassium chloride, and phosphoric acid were used to prepare the B–R buffer solution. Using 2.0 M NaOH or 2.0 M HCl, the pH values of the solutions were adjusted to range between 2.0 and 10.0. A stock solution of Mel was prepared using purified Mel powder supplied by Sigma-Aldrich Co. and a mixture of 1:1 methanol and water.

The synthesis of the nanocomposite included the use of urea (NH_2_CONH_2_), nitric acid (HNO_3_, 65%), and sulfuric acid (H_2_SO_4_, 98.0%) bought from Merck (Darmstadt, Germany). Additionally, ammonium molybdate and selenium black were acquired from Sigma-Aldrich Co. (Germany). All of the materials used in the experiments were of analytical quality. The electrochemical procedures were performed using the Metrohm-Autolab potentiostat/galvanostat system (PGSTAT128 N, The Netherlands).

The characterization of the generated g-C_3_N_4_, activated nanodiamond (ND-COOH), g-C_3_N_4_@ ND-COOH, and g-C_3_N_4_@ND-COOH@MoSe_2_ materials was conducted by the use of FTIR, XRD, SEM, and SEM-EDX techniques. The corresponding Fourier transform infrared spectrophotometry (FTIR) spectra were conducted using a PerkinElmer Spectrum 400 FTIR spectrometer (Waltham, MA, USA). The synthesized materials’ X-ray diffraction (XRD) spectra were obtained using a Bruker AXS D8 XRD diffraction meter. The morphology and structure of the synthesized materials were obtained by scanning electron microscopy images and SEM analysis using a LEO 440 SEM brand electron microscope.

### 2.2 Real Sample Preparation

Mel injection consists of Mel hydrochloride in an amount corresponding to 50 mg of Mel, along with 20 mg of povidone K12 and hydrochloric acid. The diluent solution, which is 10 mL in volume, contains water, 0.2 g of sodium citrate, 5.0 mL of propylene glycol, and 0.52 mL of ethanol. Two vials of Mel were combined, and 1.0 mL of the resulting mixture was mixed with 9.0 mL of a B–R buffer solution. For urine sample preparation, filtration was performed using a PTFE syringe filter with a membrane pore size of 0.45 μm. A specific quantity of the Mel solution was added to the urine solution to prepare a Mel-spiked urine sample. The Mel concentration in actual samples was determined by using the standard addition method.

### 2.3 Fabrication of Modified Electrodes

Alumina slurries (0.05 mm) were used to polish the glassy carbon electrode (GCE). Afterward, the GCE was rinsed with ultradistilled water and cleaned with a 1:1 ethanol–water mixture for 5 min. Afterward, the electrode was rinsed with water and left to air-dry at room temperature for 10 min. It was ensured that the composite was thoroughly mixed by using an ultrasonic laboratory bath. Around 6.0 μL of g-C_3_N_4_@ND-COOH@MoSe_2_ solution (1.0 mg mL^–1^) was applied onto the unadjusted electrode surface. Subsequently, the developed electrode was subjected to infrared heat from a lamp for 40 min to facilitate drying.

### 2.4 Synthesis of the Nanocomposite

g-C_3_N_4_@ND-COOH@MoSe_2_ was synthesized by using a hydrothermal approach. First, the calcination method, which is a simple and fast method, was used to produce g-C_3_N_4_ NPs. Typically, 1.0 g of g-C_3_N_4_ urea powder was added into the porcelain crucible and calcined in an oven at 550 °C. It was kept at this temperature for 4 h. It was then brought to room temperature spontaneously. At the end of this process, slightly yellowish g-C_3_N_4_ NPs were obtained.^30^ Second, 40 mL of a 3:1 mixture of concentrated sulfuric acid and concentrated nitric acid was added to the intact nanodiamond (0.3 g) and then sonicated in an ultrasonic bath until completely dispersed. Then, continuous magnetic stirring was done for 10 h. The prepared mixture was washed several times with distilled water and ethanol and dried at 80 °C.^31^ The hydrothermal synthesis procedure was used to produce g-C_3_N_4_–ND-COOH NPs in the next step. The synthesized g-C_3_N_4_ NPs weighed 250 mg and were dispersed in purified water. ND-COOH (50 mg) dispersed in distilled water was added to the g-C_3_N_4_ NPs, and ultrasonication was performed for 30 min. The solution consisting of g-C_3_N_4_ and ND-COOH was placed in an autoclave. The solvothermal synthesis process involved maintaining the temperature of the Teflon-lined autoclave at 180 °C for a duration time of 18 h. The Teflon was cooled to room temperature, and the resulting g-C_3_N_4_@ND-COOH NPs were carefully collected. The collected NPs were subjected to multiple using ethanol and deionized water and then dried in an oven at 80 °C.^32^ In the last step, appropriate amounts of ammonium molybdate were dissolved in deionized water and a selenium black sodium borohydride solution for the g-C_3_N_4_@ND-COOH@MoSe_2_ nanocomposite. Then, this solution was added to the above-synthesized composite and sonicated in an ultrasonic bath for 30 min to obtain a homogeneous distribution. The resulting mixture was transferred to a Teflon autoclave and incubated at 180 °C for 18 h to obtain the final product. The resulting nanocomposite was washed with deionized water and ethanol and dried in an oven at 80 °C.^33^

### 2.5 Molecular Docking Studies

The optimized geometries of the melphalan (Mel), the nanodiamond configuration (ND(C_84_)-COOH), and the drug-nanodiamond conjugate (Mel:ND-COOH) were determined using density functional theory (DFT) calculations performed with the Gaussian software (v.09).^34^ To achieve the intended objective, the aforementioned structures were designed utilizing the GaussView software (version 5.0.8).^35^ Following that, the optimization of ground-state geometries was performed utilizing a screened-hybrid functional of the Heyd, Scuseria, and Ernzerhof (HSE) level of theory.^36^ This was done in conjunction with a double-ζ-polarized 6-31G(d,p) basis set in a vacuum environment prior to considering the potential effect of the solvent. Accordingly, the solvent effects were implicitly accounted for by employing the Integral Equation Formalism of the Polarizable Continuum Model (IEFPCM) with a dielectric constant of water (ε_0_ = 80.4).^37^ After the necessary preliminary procedures were performed, the local minima were confirmed by conducting vibrational frequency calculations. The absence of an imaginary frequency provided definitive proof that the geometry optimization processes were successfully carried out. The resulting output files were then visualized using either Chemcraft (version 1.8)^38^ or GaussView. The molecular docking analysis of the complexes Mel–P53 and Mel:ND-COOH-P53 was performed using the AutoDock Vina extension integrated into the SAMSON software 2023 R1 (https://www.samson-connect.net/). Subsequently, visualization studies were conducted using SAMSON, UCSF ChimeraX (v.1.6.1),^39^ or Discovery Studio Visualizer 2021 (client version; Accelrys Software Inc., San Diego, CA, USA). The X-ray crystal structure of P53 (PDB ID: 6SHZ) was obtained from the Protein Data Bank. Sequentially, only chain A was chosen for the P53 analysis followed by additional pretreatment procedures involving the removal of water molecules and ligands and incorporating charges and hydrogen atoms. To build the search space within the receptor molecule, the Autogrid tool was utilized to create a 3-D grid box with dimensions of 45 × 45 × 45 Å. The investigation of possible ligand conformations within a certain spatial domain was subsequently conducted using the Lamarckian genetic algorithm. The chosen algorithmic strategy was designed to explore conformational space while minimizing computational expenses effectively. The calibration of the parameters in the docking simulation was conducted in such a way as to produce a total of 100 unique conformations in each run. Each of these docking conformations underwent a maximum of 250,000 assessments, which were carried out to guarantee the reliability and precision of the simulation outcomes. In this comprehensive molecular docking protocol, an in-depth analysis was undertaken to examine the binding affinities and interacting behaviors of Mel, ND-COOH, and P53. The findings of this careful analysis offer valuable molecular insights that complement the electrochemical investigations carried out in this inquiry.

## 3 Results and Discussion

### 3.1 Characterizations of the Composite

#### 3.1.1 XRD Analysis

**Figure 2:**
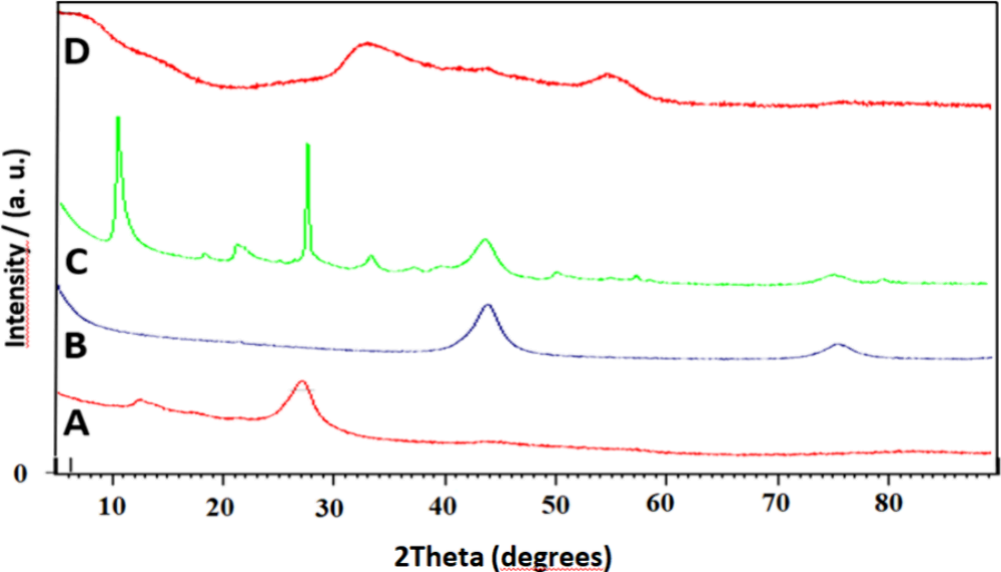
XRD pattern of g-C_3_N_4_(A), ND-COOH (B), g-C_3_N_4_@ND-COOH (C), and g-C_3_N_4_@ND-COOH@MoSe_2_ (D) nanomaterials.

Figure 2 shows the XRD pattern of the materials g-C_3_N_4_(*a*), ND-COOH (b), g-C_3_N_4_@ND-COOH (c), and g-C_3_N_4_@ND-COOH@MoSe_2_ (d).

The XRD pattern of g-C_3_N_4_ (JCPDF No. 87-1526) has characteristic interplanetary peaks at 2θ = 27.5 and 13.1° corresponding to the diffraction planes (0 0 2) and (1 0 0). These peaks correspond to the stacking of the conjugated aromatic structure and the in-plane repeating units of the interplanetary tri-s-triazine unit.^40−43^ In the ND-COOH diffraction pattern, the diffraction peaks corresponding to planes (1 1 1) and (2 2 0), respectively, show the diffraction peaks at 2θ = 44.7 and 75.4°.^31,44^ X-ray diffraction patterns of the g-C_3_N_4_@ND-COOH composite are given in Figure 2C. In the XRD model of g-C_3_N_4_@ND-COOH, C_3_N_4_ peaks were detected at 10.7, 27.9, 43.8, and 75.6°. Also, as with the ND-COOH structure (2B), g-C_3_N_4_@ND-COOH (C) had a diffraction peak of 2θ = 43.66°.^31,40,41,43−45^ When the spectrum of g-C_3_N_4_@ND-COOH NPs is examined, it is seen that characteristic peaks of g-C_3_N_4_ and ND-COOH NPs are also obtained at higher peak intensities. With the addition of MoSe_2_ and g-C_3_N_4_@ND-COOH@MoSe_2_ hybrid, prominent diffraction peaks of MoSe_2_ were obtained, consistent with those of standard MoSe_2_. MoSe_2_ shows a hexagonal structure and characteristic peaks at 31.42, 37.88, and 55.92° (JCPDS No. 29-0914).^33,46,47^ In addition, no significant peak is observed due to the weak crystal structure of MoSe_2_ in the composites. XRD patterns seem to be in good agreement with the literature data.

#### 3.1.2 FTIR Analysis

The functional groups present in the g-C_3_N_4_ (Figure S1A), ND-COOH (Figure S1B), g-C_3_N_4_@ND-COOH (Figure S1C), and g-C_3_N_4_@ND-COOH@MoSe_2_ (Figure S1D) samples were investigated using FTIR spectroscopy in the range of 450–4000 cm^–1^.

The heterocyclic structure of g-C_3_N_4_ (aromatic) is evident in Figure S1A, where many distinct peaks are seen within the 1200–1650 cm^–1^ range. These peaks correspond to the stretching vibrations of the C–N and C=N bonds in the CN aromatic repeating units. The absorption band found in g-C_3_N_4_ is attributed to the vibrational mode of the triazine ring, which serves as a distinctive property of this material. The observed peak at a wavenumber of 3188 cm^–1^ may be attributed to the vibrational modes associated with stretching the N–H bond. The confirmation of nanodiamond functionalization is further shown by the peaks seen as a result of different functional groups on ND-COOH during the characterization of their structural–functional characteristics using FTIR.^42,43^ Moreover, when the FTIR spectrum of g-C_3_N_4_@ND is compared with the FTIR spectrum of ND-COOH (Figure S1B) and g-C_3_N_4_ (Figure S1A), the similarities between the spectrum clearly reveal the surface functional groups of the structure g-C_3_N_4_@ND-COOH (3C). The broad peak around 3180 cm^–1^ indicates an enhanced contribution of g-C_3_N_4_. These FTIR spectra provide information about the successful synthesis and chemical structure of the hybridization (3D) of MoSe_2_ with g-C_3_N_4_@ND-COOH nanoparticles, whereas the characteristic peaks of g-C_3_N_4_@ND-COOH in the composite structure prove the presence of the structure.

#### 3.1.3 FE-SEM and SEM-EDX Analysis

**Figure 3:**
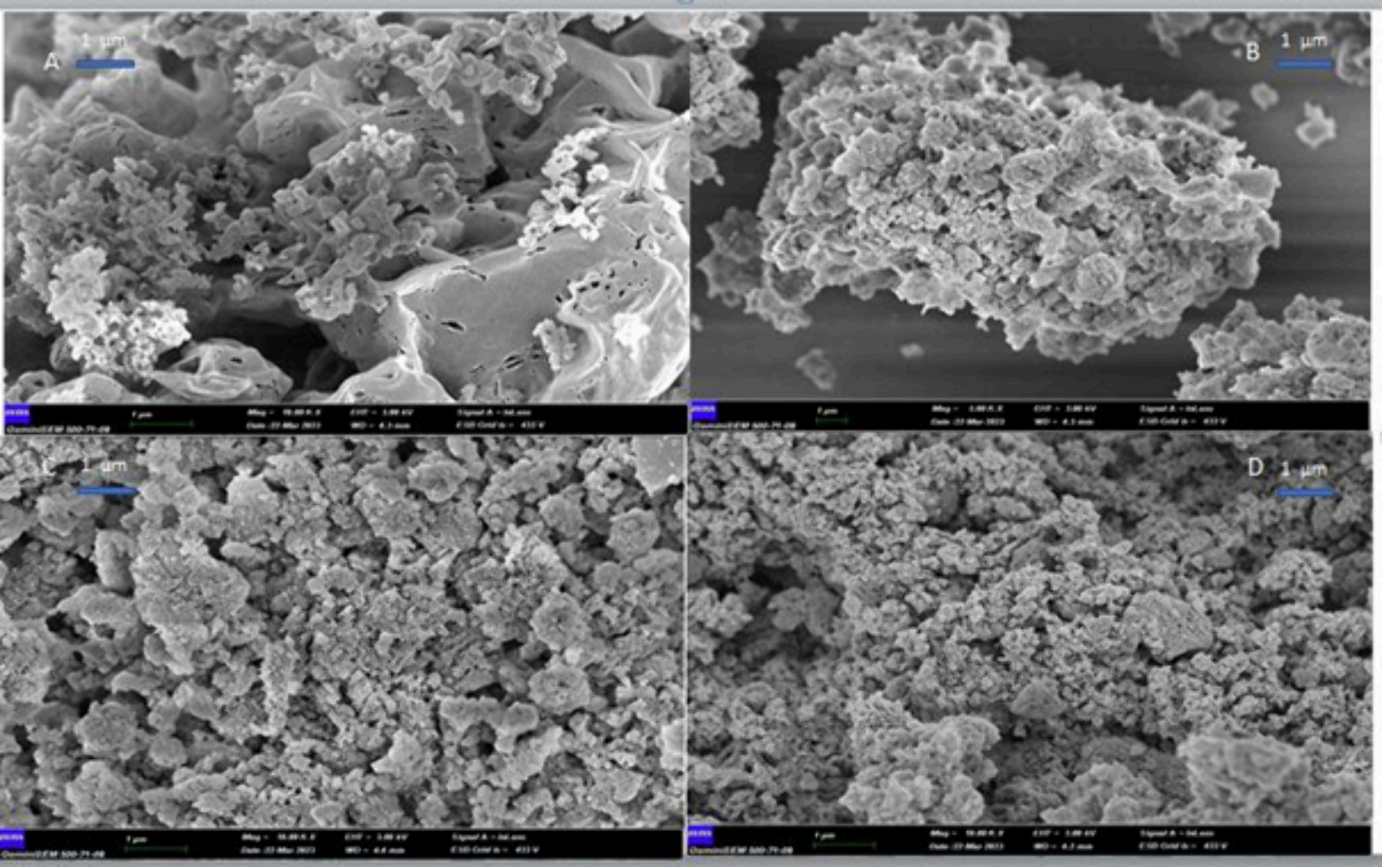
SEM image of g-C_3_N_4_(*a*), ND-COOH (b), g-C_3_N_4_@ND-COOH (c), and g-C_3_N_4_@ND-COOH@MoSe_2_ (d) nanomaterials.

SEM images at different magnifications (5.0 KX–10.0 KX) using FE-SEM to examine the surface morphology and shape of the synthesized g-C_3_N_4_, ND-COOH, g-C_3_N_4_@ND-COOH, and g-C_3_N_4_@ND-COOH@MoSe_2_ are shown in Figure 3A–D, respectively.

As shown in Figure 3A, g-C_3_N_4_ NPs appear to have a porous structure after calcination. Figure 3B,C present images of ND-COOH and g-C_3_N_4_@ND-COOH NPs. The results show that the formation of a hybrid structure is possible by combining the structural morphology of g-C_3_N_4_@ND-COOH with the hydrothermal method when g-C_3_N_4_ is added. FE-SEM images of g-C_3_N_4_@ND-COOH demonstrate the effective interconnection of ND-COOHs on the surface of g-C_3_N_4_, showing an irregular nanoparticle-like structure. Then, it was observed that the structure consisting of multiple layers of MoSe_2_ was densely coated on the surface of the g-C_3_N_4_@ND-COOH.

**Figure 4:**
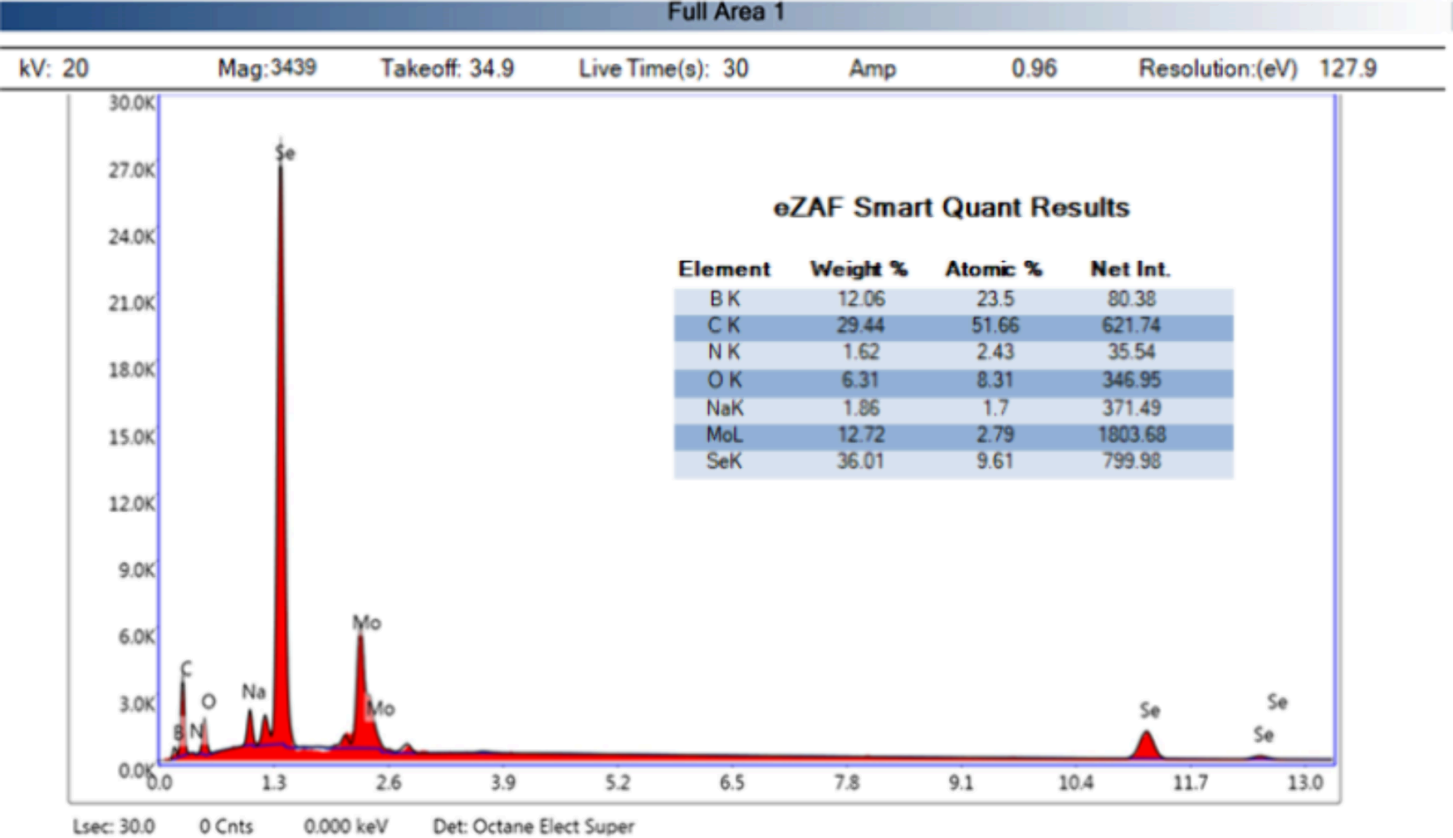
SEM-EDX spectrum of g-C_3_N_4_@ND-COOH@MoSe_2_.

In addition, EDX analysis was performed to determine the elements contained in the g-C_3_N_4_@ND-COOH@MoSe_2_ material obtained in Figure 4. Elemental mapping images reveal the presence of B, O, C, N, Se, and Mo elements and the approximate distribution of each g-C3N4@ ND-COOH@MoSe2 composite element. FE-SEM and EDX results show the successful recovery of g-C_3_N_4_@ND-COOH@MoSe_2_ NPs.

As the results of the characterizations (FTIR, XRD, FE-SEM, SEM-EDX) are in agreement, it shows that g-C_3_N_4_@ND-COOH@MoSe_2_ nanostructures were successfully obtained.

### 3.2 Electrochemical Studies of g-C_3_N_4_@ND-COOH@MoSe_2_

Differential pulse voltammetry (DPV) was utilized to evaluate the electrochemical performance of both unmodified and modified electrodes for detecting 0.1 mmol L^–1^ Mel in 0.1 M Britton–Robinson (B–R) buffer at pH 2.0. This investigation is illustrated in Figure S2. The initial measurement was carried out using the bare GCE followed by measurement of the modified electrode coated with g-C_3_N_4_@ND-COOH@MoSe_2_. The results depicted in Figure S2 illustrate that when Mel solution (0.1 mmol L^–1^) was introduced to the electrochemical medium, a noticeable anodic peak current around 0.8 V was obtained. This result highlights the significant role of the modified electrode in facilitating the oxidation of the target analyte. The presence of additional active sites or catalytic species on the electrode surface leads to an observed oxidation current and a peak intensity elevation. Notably, the current value showed an impressive approximate 6-fold increase compared to the unmodified electrodes.

**Figure 5:**
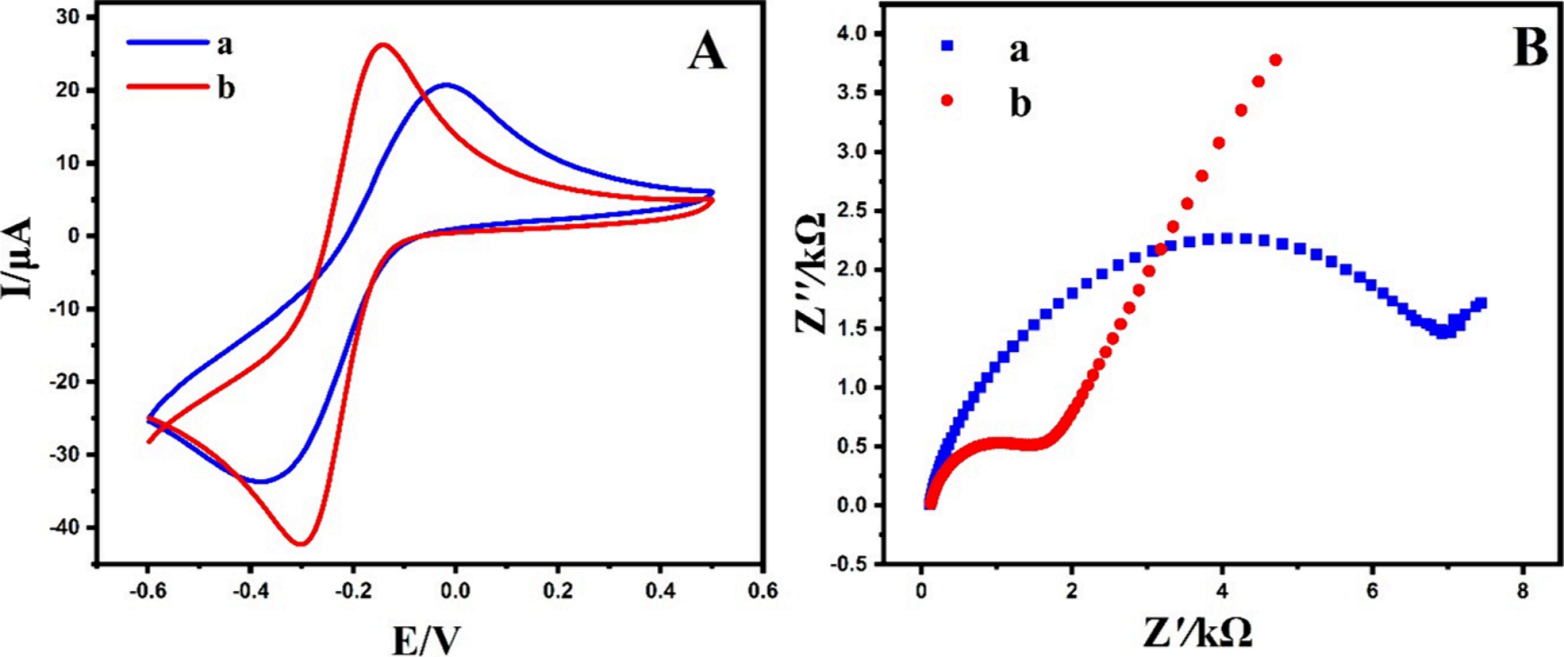
(A) CVs of bare GCE (a) and g-C_3_N_4_@ND-COOH@MoSe_2_/GCE (b) at a scan rate of 50 mV/s in 0.1 M KCl solution containing 5.0 mM K_3_[Fe(CN)_6_]. (B) Nyquist plots of EIS of the unmodified GCE (a) and g-C_3_N_4_@ND-COOH@MoSe_2_/GCE (b).

To assess the electrochemical behavior of the bare and modified electrodes, cyclic voltammetry (CV) was employed. Figure 5A illustrates the CVs obtained from the bare GCE and the g-C_3_N_4_@ND-COOH@MoSe_2_/GCE using a scan rate of 50 mVs^–1^ in a 0.1 M KCl solution containing 5.0 mmol L^–1^ K_3_[Fe(CN)_6_]. The modified GCE exhibited a higher oxidation peak current, resulting in a lower Δ*E*_p_ value of 0.14 V compared to that of the bare electrode (0.3 V). This enhancement can be attributed to the accelerated electron transfer rate and increased electrocatalytic activity facilitated by modifying the GCE.

Furthermore, the conductive properties of the modified electrode were assessed by performing electrochemical impedance spectroscopy (EIS) measurements, which were conducted across a frequency range of 10 kHz to 0.1 Hz at a potential of 0.1 V for both the bare electrode and the g-C_3_N_4_@ND-COOH@MoSe_2_/GCE. As depicted in Figure 5B, the Randles equivalent circuit (*R*_ct_) value for g-C_3_N_4_@ND-COOH@MoSe_2_/GCE was 2013.8 Ω, which was higher compared to that for the bare electrode (10,976 Ω). The decrease observed in the load transfer resistance suggests that incorporating g-C_3_N_4_@ND-COOH@MoSe_2_ enhances the electron transfer kinetics on the electrode. This improvement can be ascribed to the strong conductivity of the modifier, g-C_3_N_4_@ND-COOH@MoSe_2_.

**Figure 6:**
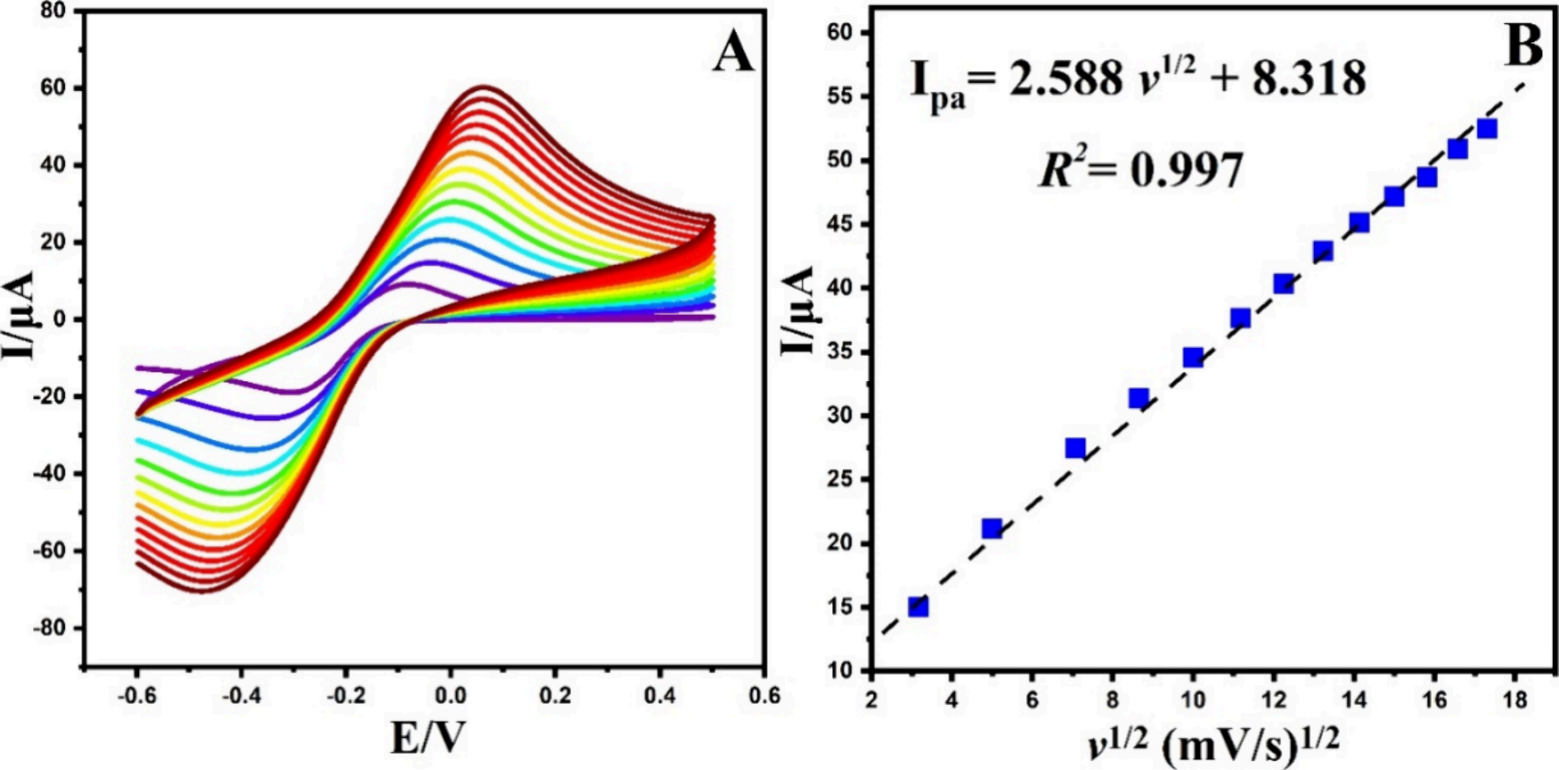
(A) CVs of unmodified electrode at different scan rates (10.0–300.0 mV/s) in 0.5 mM K_3_[Fe(CN)_6_] containing 0.1 M KCl. (B) The plot of *I*_pa_ vs *v*^1/2^.

**Figure 7:**
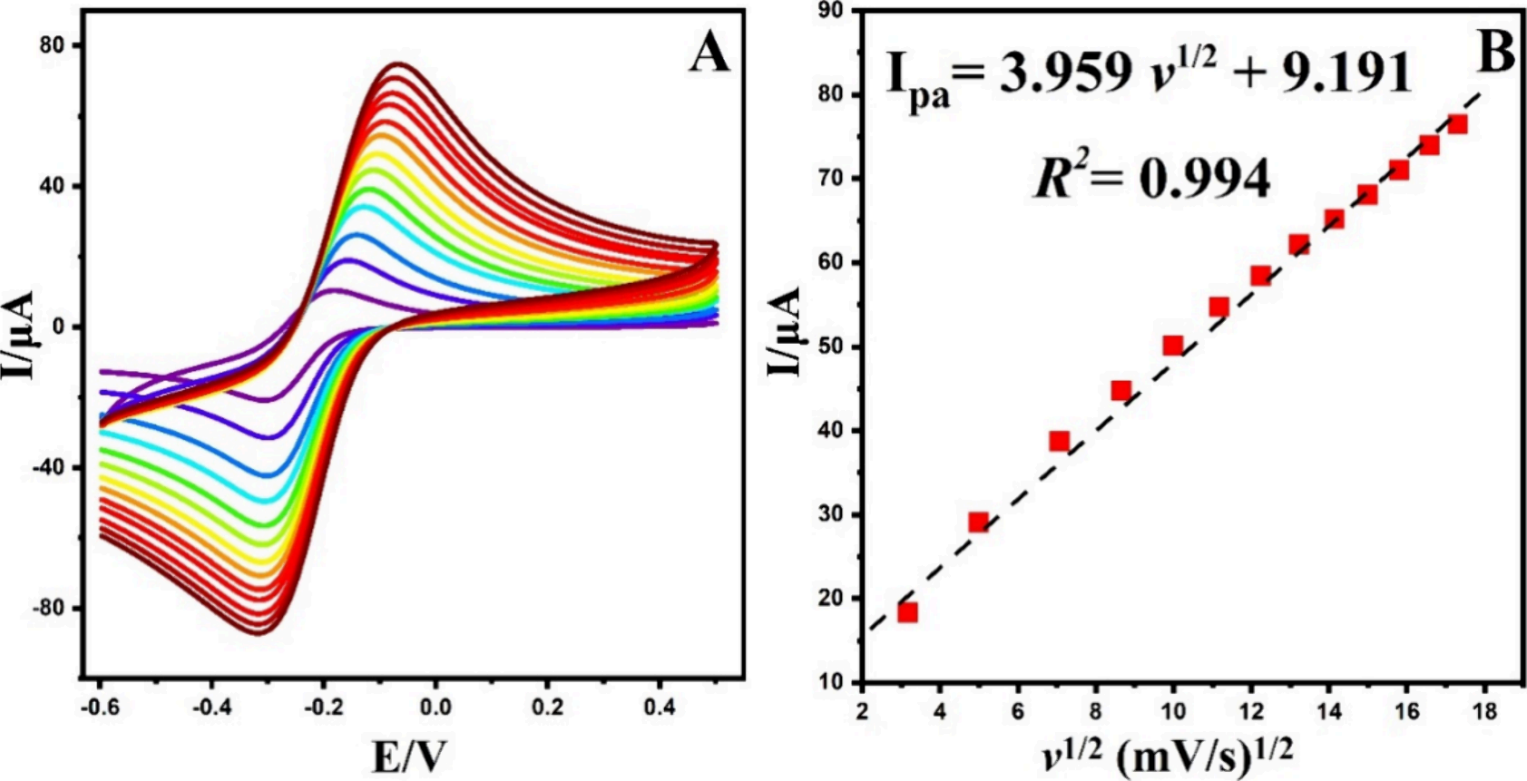
(A) CVs of the g-C_3_N_4_@ND-COOH@MoSe_2_/GCE electrode at different scan rates (10.0–300.0 mV/s) in 0.5 mM K_3_[Fe(CN)_6_] containing 0.1 M KCl. (B) The plot of *I*_pa_ vs *v*^1/2^.

The electroactive surface area is essential in developing electrodes, as it significantly impacts their performance. By increasing the surface area, a large number of sites for electrochemical reactions can be obtained, accordingly enhancing the electrochemical characteristics. In this study, the electrochemically active surface area of both the GCE and g-C_3_N_4_@ND-COOH@MoSe_2_/GCE was estimated in a solution consisting of 0.1 M B–R buffer and 5.0 mmolL^–1^ K_3_[Fe(CN)_6_]. The Randles–Sevcik equation (eq 1) analyzed the plot’s slope between the square root scan rate vs peak current (Figures 6 and 7).
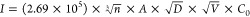
1

The calculated responsive surface areas of the GCE and g-C_3_N_4_@ND-COOH@MoSe_2_/GCE electrodes were 0.074 and 0.113 cm^2^, respectively. These results indicate that the adjusted electrode has a larger surface area than the unmodified GCE. The improvement observed can be ascribed to the elevated conductivity and the collaborative impact of the integrated materials.

### 3.3 Optimization of the Developed Electrode

Further investigations are crucial to optimizing the modified electrode, focusing on factors such as the suitable electrolyte, composite concentration and amount, and electrolyte pH level. To establish optimal working conditions, selecting a suitable medium is crucial. Figure S3A illustrates the evaluation of various electrolytes, including HCl, KCl, NaOH, and PBS, to identify the most favorable working conditions. Notably, the B–R buffer demonstrated superior performance, providing an ideal environment for the experiment (Figure S3A). To identify the optimal current effectiveness, it is necessary to ascertain the suitable concentration. The influence of varying concentrations of g-C_3_N_4_@ND-COOH@MoSe_2_ on the electrode surface was investigated within the 0.1–2.0 mg/mL range (Figure S3B). Among the tested concentrations, a remarkable improvement in the oxidation response of the desired analytes was observed at a concentration of 2.0 mg/mL of the g-C_3_N_4_@ND-COOH@MoSe_2_ composite. As the concentration of g-C_3_N_4_@ND-COOH@MoSe_2_/GCE increased from 0.1 to 2.0 mg/mL, a significant enhancement in the current percentage was observed. This enhancement can be attributed to the availability of more adsorption-active sites and an increased surface area. However, at larger concentrations (beyond 2.0 mg/mL), a noticeable decrease in current may occur because of the aggregation or excessive expansion of available interaction sites.

In addition, the impact of the quantity of the g-C_3_N_4_@ND-COOH@MoSe_2_ composite on the performance and sensitivity of the electrode was examined within the 4.0–8.0 μL range (Figure S3C). As shown in Figure S3C, the anodic current reached its maximum when 6.0 μL of the composite was used. Nevertheless, with larger amounts used, a noticeable decline in the oxidation peak current was observed, likely ascribed to the reduced attachment of the modifier layer on the electrode surface.

### 3.4 Impact of pH and Scan Rate

**Figure 8:**
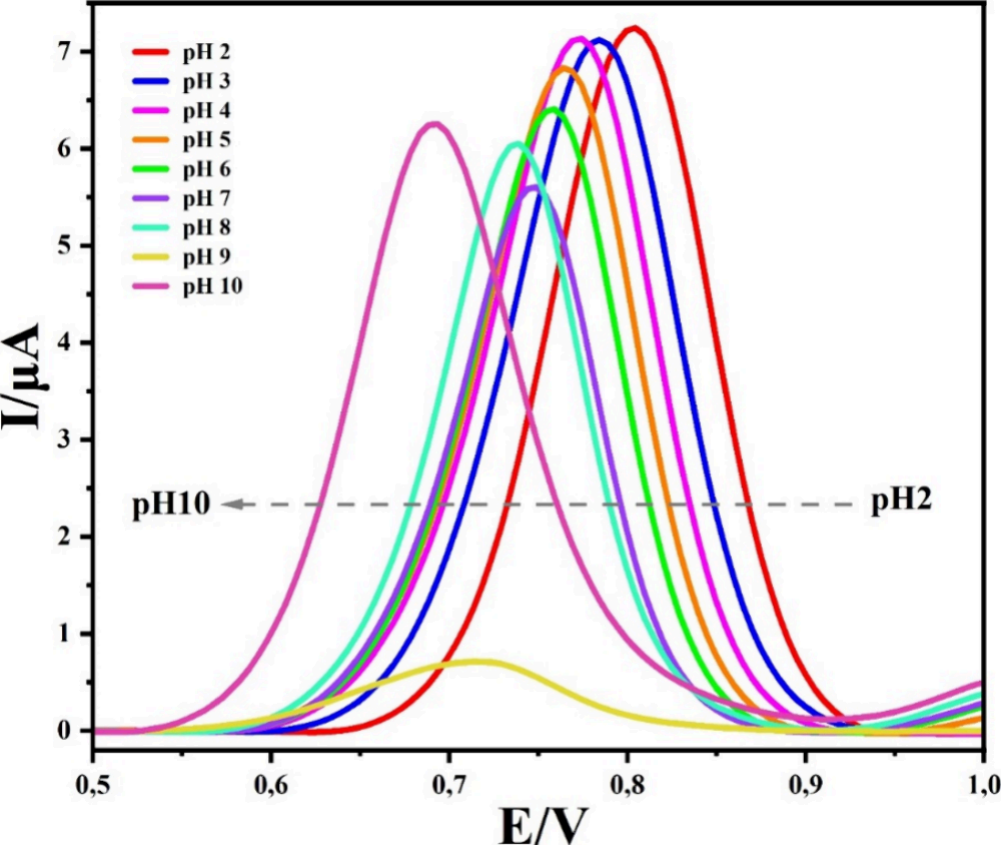
DPVs of 0.1 mM Mel were observed at different pH levels.

The impact of the medium’s pH was investigated in the medium of a 0.1 M B–R buffer, with pH values ranging from 2.0 to 10.0. As illustrated in Figure 8, Mel’s oxidation peak current decreased as the pH value rose from 2.0 to 10.0. Consequently, a basic electrolyte with a pH of 2.0 was chosen for subsequent experiments.

Moreover, Figure 8 demonstrates that the oxidation peak of Mel displayed a change toward negative-shifted potential with higher pH levels, indicating an irreversible process and electrochemical response of g-C_3_N_4_@ND-COOH@MoSe_2_/GCE depending on the pH of the supporting electrolyte.^48^

**Figure 9:**
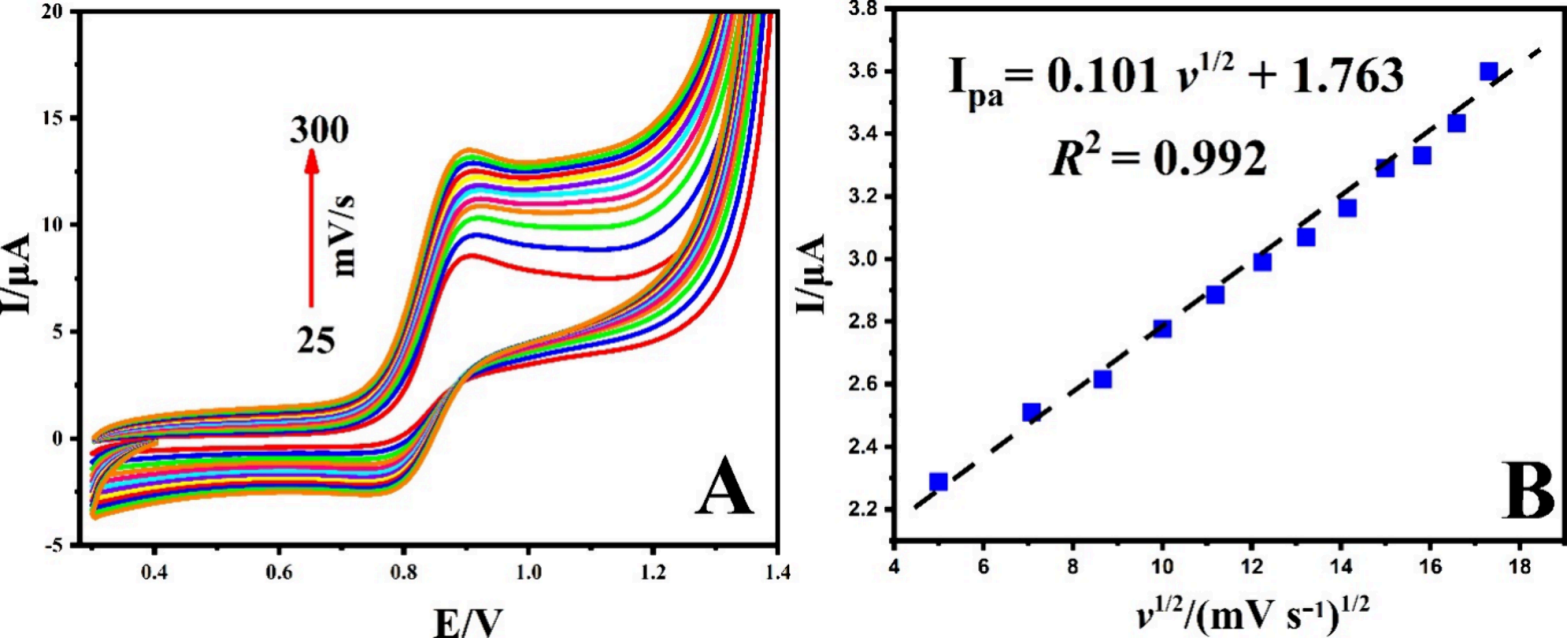
(A) CVs of g-C_3_N_4_@ND-COOH@MoSe_2_/GCE. (B) Peak current and the square root of the scan rate.

To investigate the electrooxidation character of Mel on g-C_3_N_4_@ND-COOH@MoSe_2_/GCE, CV tests were conducted at different scan rates (ranging from 10.0 to 300.0 mV s^–1^) under optimized conditions. The corresponding outcomes are presented in Figure 9A. With an increase in the scanning rate, the oxidation peak of Mel exhibited a shift toward a more negative potential accompanied by a reduction in the peak current (Figure 9A).

Additionally, as can be seen in Figure 9B, a linear correlation was observed between the peak current and the square root of the scan rate (*v*^1/2^), expressed by the following equation:
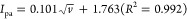


This finding confirms that the electrocatalytic oxidation of Mel follows a diffusion-controlled process.

### 3.5 Analytical Application

**Figure 10:**
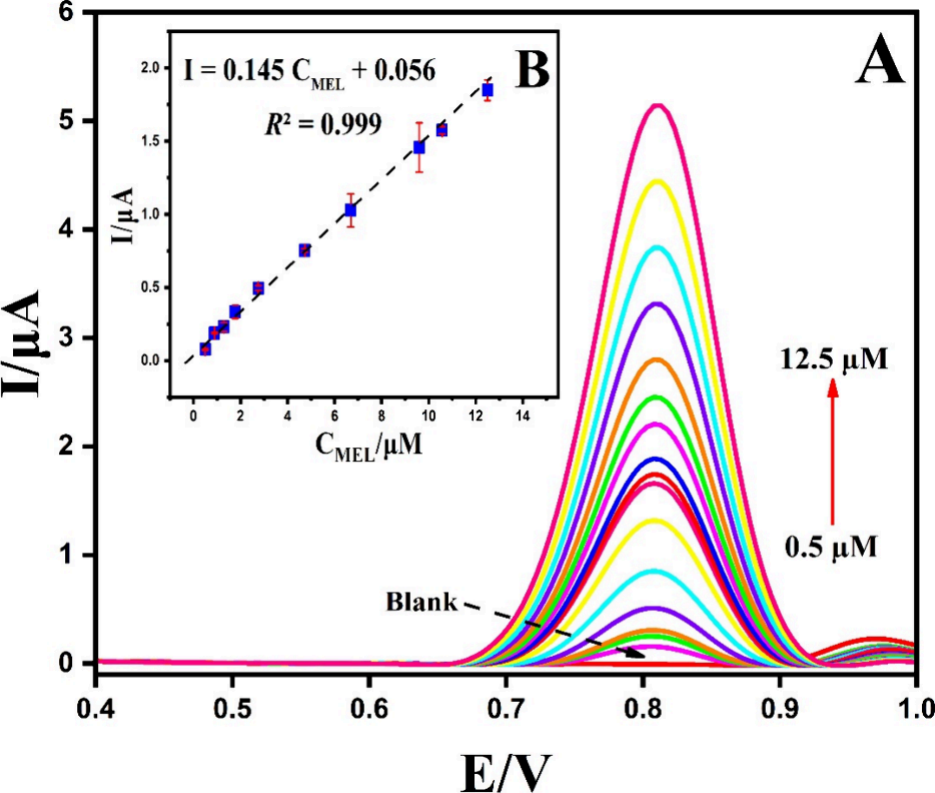
(A) DPVs of different concentrations of Mel from 0.5 to 12.5 μM. (B) Linear dependence of peak currents and Mel concentrations.

By employing differential pulse voltammetry (DPV) under optimal conditions, a calibration curve was constructed across a broad concentration range of 0.5–12.5 μM, as shown in Figure 10.

The DPV current peak significantly increased as the Mel concentration rose, primarily as a result of Mel’s oxidation process. The relationship between the peak current and Mel concentrations displayed linearity, represented by the linear equation I = 0.145 *C*_Mel_ + 0.056 (*R*^2^=0.999). Based on the calibration curve, the limit of detection (LOD) was determined using the 3σ (standard deviation)/slope method,^49^ considering three times the standard deviation of the blank measurement, resulting in an LOD of 0.03 μM. Additionally, the presence of only one linear equation implies the absence of kinetic limitations, indicating efficient electron transfer that successfully overcomes any kinetic barriers. These findings provide strong evidence that the g-C_3_N_4_@ND-COOH@MoSe_2_/GCE platform holds great promise for the electrochemical detection of Mel.

### 3.6 Selectivity, Repeatability, and Reproducibility

**Figure 11:**
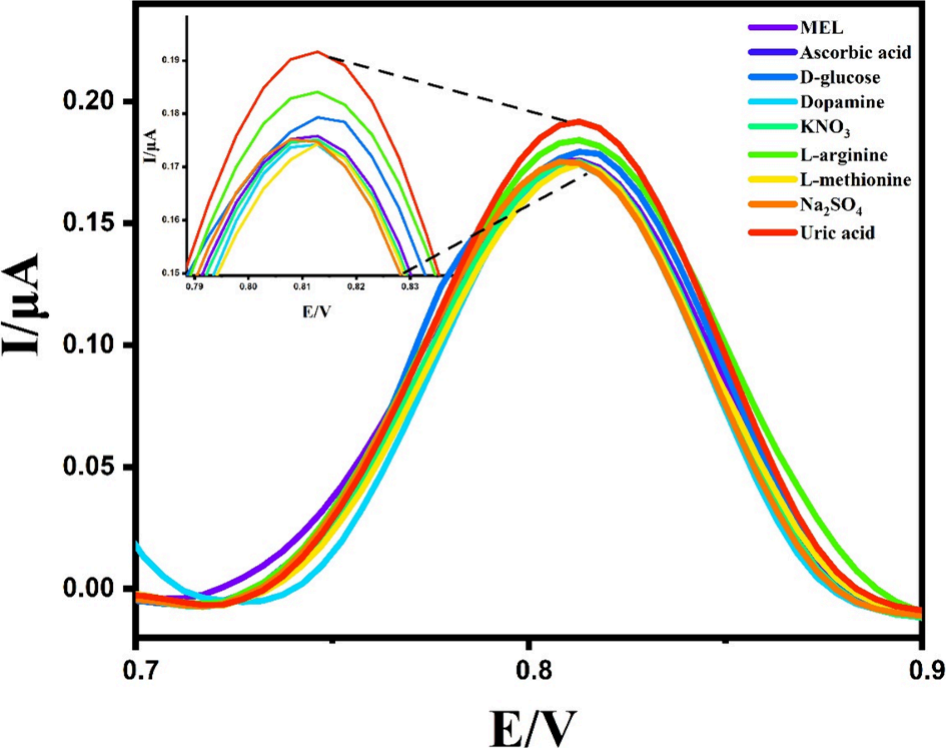
Selectivity of Mel at pH 2.

The primary goal in the design of electrochemical sensors is typically to selectively distinguish the target analyte in the environment of other interfering species. Therefore, the criticality of analytical specificity as a fundamental characteristic significantly impacts the precision of the analysis. This study aimed to examine the specificity of Mel in the presence of various bioactive compounds, including l-arginine and glucose, potassium chloride, sodium hydroxide, and potassium nitrate, using DPV under an optimal environment. When 0.1 μM of Mel, as depicted in Figure 11, was incubated alongside a 1000-fold higher concentration of different coexisting species, there was no significant change in the signal compared to the pure Mel target. The developed electrode exhibited high selectivity, as indicated by the relative standard deviation (RSD) of 2.0%. Nevertheless, a minor change in the anodic potential of Mel was noted, possibly due to the elevated ionic strength of the solution and potential interactions among the components.

The procedure was repeated 15 times to obtain successive responses to assess the repeatability of the g-C_3_N_4_@ND-COOH@MoSe_2_/GCE electrode (Figure S4A). The g-C_3_N_4_@ND-COOH@MoSe_2_/GCE electrode demonstrated excellent repeatability, as made evident by the calculated RSD of 2.43%. Furthermore, the reproducibility of the modified electrode was evaluated by fabricating nine separate g-C_3_N_4_@ND-COOH@MoSe_2_/GCE electrodes using an identical procedure (Figure S4B). The RSD was calculated to be 3.77%, indicating the reproducibility of the modified electrode.

### 3.7 Analysis of Actual Samples

To validate the applicability of the modified electrode, the detection of Mel was carried out in human urine and pharmaceutical (injection) samples. The standard addition method was employed to determine the recovery of Mel from known concentrations by using the advanced sensing method. The recovery rates ranged from 97.2 to 103.1% for urine and from 98 to 105% for injection (Table 1). These findings demonstrate that the electrochemical sensing strategy developed in this study is appropriate for the accurate detection of Mel in the actual samples.

**Table 1:** Determination of Mel with g-C_3_N_4_@ND-COOH@MoSe_2_/GCE in Real Samples

**sample**	**added (μM)**	**found (μM)**	**recovery (%)**
urine	0.75	1.35	103.1
	0.90	1.48	100.7
	3.00	3.32	97.23
injection	1.50	1.62	98.76
	6.00	6.67	105.8
	8.00	7.28	101.5

### 3.8 Molecular Docking Studies

After the docking process, a thorough analysis was conducted on the binding poses to determine the most likely orientation of Mel and Mel:ND-COOH within the binding pocket of P53. The binding affinity values were calculated in kcal/mol, which facilitated a comparative analysis of the binding efficacy between Mel (*or* Mel:ND-COOH) and P53. The binding affinity of Mel with P53 was found to be −5.0 kcal/mol, whereas the modeled covalent conjugate Mel-nanodiamond-COOH exhibited a significantly enhanced binding affinity of −10.9 kcal/mol, thereby elucidating the superior binding propensity of the ND-COOH conjugate. Further analysis was undertaken to decipher the key interactions, such as hydrogen bonding, π–π stacking, and hydrophobic interactions, between the ligands and the amino acid residues within the binding pocket of P53. As previously indicated, visualization and analysis of the docking results were performed using the SAMSON software, and the interaction maps were generated to visually represent the key interactions contributing to the binding affinity (Table 2).

**Table 2:**
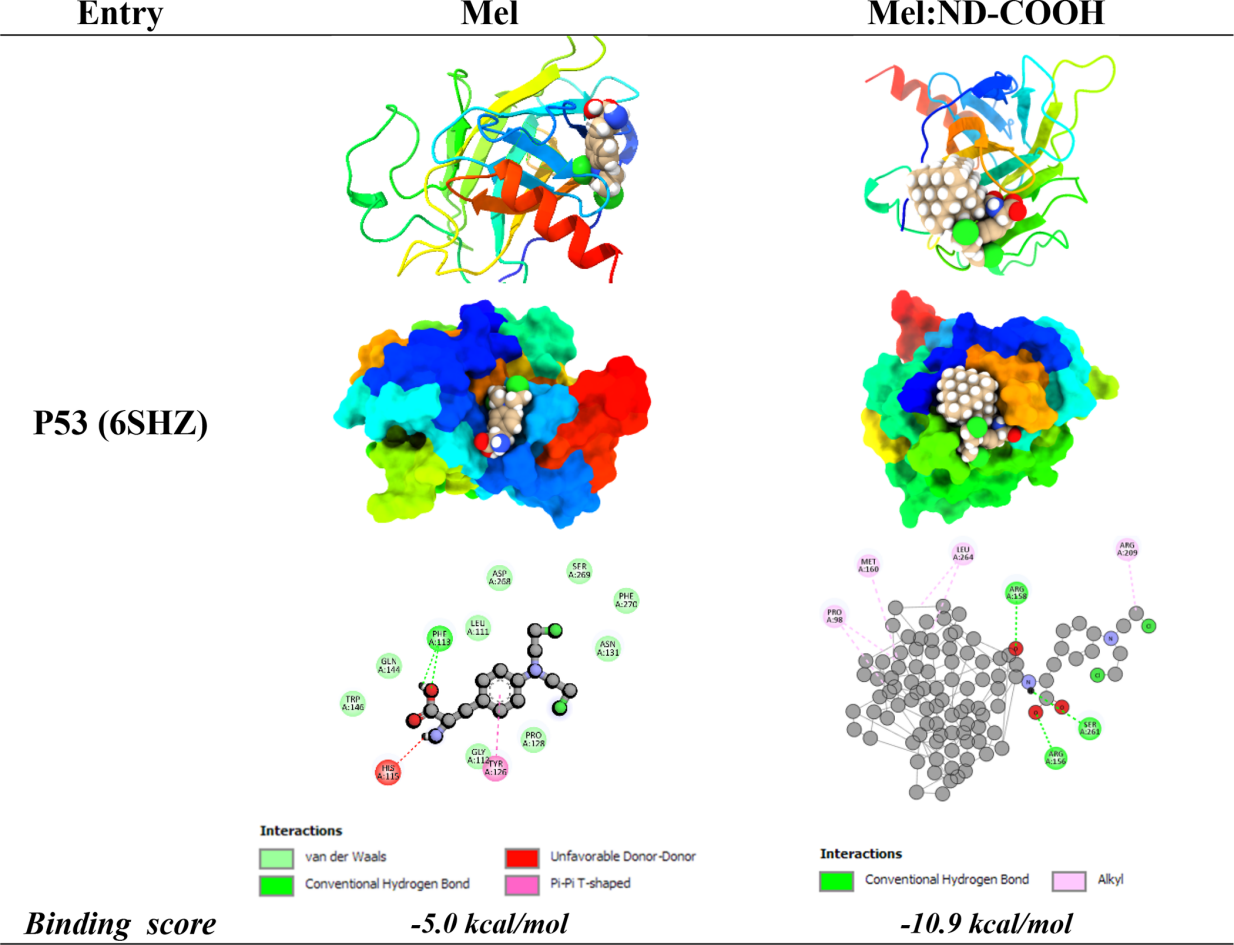
Binding Poses and Residue Interactions of Mel and Mel:ND-COOH with P53 (PDB ID: 6SHZ)

Regarding Mel–P53 interactions, the docking studies identified significant intermolecular contacts. For instance, two conventional hydrogen bonds were noticed between the atom H of Mel and the oxygen atom of PHE113 in P53 and reciprocally between the hydrogen atom of PHE113 and the oxygen atom of Mel, solidifying the complex formed between Mel and P53. Additionally, a nonconventional π–π T-shaped interaction was observed between Mel and TYR126 in P53, further stabilizing the Mel–P53 complex and enhancing the binding affinity, which was calculated to be −5.0 kcal/mol. This negative binding score, indicative of spontaneous interaction, underscores the potential efficacy of molecular interactions’ potential efficacy. On the other hand, the molecular docking analysis also illuminated an unfavorable interaction within the Mel–P53 complex. Specifically, a donor–donor clash was observed between melphalan’s hydrogen atom (H) and a hydrogen atom (HE2) of the HIS115 residue in P53. The interaction was recognized as unfavorable, with a distance of 1.96498 Å, presenting a less favorable scenario that could potentially hinder the optimum binding between Mel and P53. Unfavorable donor–donor interactions generally signify a situation where two hydrogen atoms are in close proximity, which can lead to repulsion due to similar electronic characteristics, potentially affecting the molecular complex’s stability. This unfavorable interaction underlines the importance of analyzing the molecular docking results to identify the favorable interactions that contribute to binding affinity and the unfavorable interactions that might pose challenges or offer insights for further optimization. Recognizing such unfavorable interactions is crucial as it provides a nuanced understanding of the molecular interactions at play and may guide subsequent structural modifications to either the sensor design or the molecular entities involved, aimed at mitigating such unfavorable interactions and enhancing the sensor’s binding affinity and overall performance.

In contrast, the complex formed by Mel:ND-COOH-P53 demonstrated a variety of intermolecular interactions, hence strengthening the overall stability of the complex. The analysis unveiled multiple typical hydrogen bond interactions, specifically between the hydrogen atom of Mel:ND-COOH and the oxygen atom of SER261, as well as between ARG156 and Mel:ND-COOH and between ARG158 and Mel:ND-COOH. In addition, a variety of hydrophobic contacts were observed in the Mel:ND-COOH-P53 complex, specifically alkyl–alkyl interactions involving Mel:ND-COOH with MET160, LEU264, ARG209, and PRO98. These interactions play a crucial role in enhancing the stability of the complex. The Mel:ND-COOH-P53 complex has a notable binding affinity of −10.9 kcal/mol. This suggests a greater level of interaction effectiveness in comparison with the Mel–P53 complex.

This series of molecular interactions corroborates the heightened detection capabilities of the sensor, underscored by the superior binding affinity exhibited by ND-COOH, thereby accentuating its appropriate selection in sensor design. These molecular docking results furnish compelling insights into the specificity and strength of Mel’s and Mel:ND-COOH’s interaction with P53, fostering a more profound understanding of its molecular mechanism in a therapeutic context. The observed molecular interactions and the binding scores lend credence to the potential of the Mel:ND-COOH conjugate in enhancing the electrochemical sensor’s efficiency for Mel detection, thus contributing significantly toward the larger goal of advancing cancer therapeutic strategies and monitoring methodologies.

## 4 Conclusions

We have successfully developed and analyzed an innovative electrochemical sensor utilizing g-C_3_N_4_@ND-COOH@MoSe_2_/GCE to showcase the benefits of employing these materials for detection purposes and expanding the range of applications for nanoparticle-based materials. The fabricated structure underwent comprehensive characterization through FTIR, SEM, and XRD to confirm the accomplished fabrication of g-C_3_N_4_@ND-COOH@MoSe_2_/GCE.

Under optimized conditions, the g-C_3_N_4_@ND-COOH@MoSe_2_/GCE demonstrated excellent selectivity under the conditions of different interferents as well as remarkable repeatability and reproducibility. The electrochemical sensor demonstrated a broad linear concentration range of 0.5–12.5 μM, along with a remarkable sensitivity and low detection limit (LOD) of 0.03 μM.

To validate the reliability of our developed sensing strategy, g-C_3_N_4_@ND-COOH@MoSe_2_/GCE was used to determine Mel in actual samples, including pharmaceutical products. The obtained results showed satisfactory recovery rates ranging from 97.2 to 103.1% for urine and 98 to 105% for injection and low relative standard errors, indicating the accuracy and precision of the sensor.

In general, our study not only presents a viable methodology for the identification of Mel but also proposes prospective applications for the determination of other antiviral medications utilizing g-C_3_N_4_@ND-COOH@MoSe_2_/GCE. The practicality of implementing this sensing platform is further enhanced by its simplicity. This study presents novel opportunities for the advancement of practical techniques in the identification of diverse antiviral medications.

The molecular docking revealed a binding affinity of −5.0 kcal/mol between Mel and p53, wheresa a significantly more favorable binding score of −10.9 kcal/mol was observed for the interaction between ND-COOH and Mel. These findings underscore the potential of ND-COOH in enhancing the electrochemical sensor’s selectivity and sensitivity toward Mel detection. Moreover, the enhanced binding affinity elucidated through molecular docking substantiates the choice of ND-COOH in the sensor design, aligning with the broader objective of achieving precise, rapid, and cost-effective Mel detection. These molecular interaction analyses, coupled with electrochemical sensor development, pave the way for a more nuanced understanding of Mel’s molecular interactions and their detection, fostering the overarching goal of advancing cancer therapeutics through integrated analytical and molecular approaches.

## Data Availability

The authors confirm that the data supporting the findings of this study are available within the Supporting Information file.

## Supporting Information Available

Additional experimental details (DPVs) and FTIR spectra of studied nanocomposites (PDF)
