# Supplementary material for: Electrochemical Detection of Melphalan in Biological Fluids Using a g-C3N4@ND-COOH@MoSe2 Modified Electrode Complemented by Molecular Docking Studies with Cellular Tumor Antigen P53
Source: ACS Omega. 2024 Apr 30;9(19):21058–70. doi: 10.1021/acsomega.4c00558 (PMC11097377; doi:10.1021/acsomega.4c00558)
Supplement: Supplementary file 1 — ao4c00558_si_001.pdf [file ao4c00558_si_001.pdf]

Supplementary Information

Electrochemical Detection of Melphalan in Biological Fluids using g-C_3N_4@ND-COOH@$MoSe_2$ Modified Electrode Complemented by Molecular Docking Studies with Cellular Tumor Antigen P53

Nevin Erk[a,*], Gülbin Kurtay[b,*], Wiem Bouali[a,c], Zeyneb Gülsüm Sakal[a,c], Asena Ayşe Genç[a,c], Zeliha Erbaş[d,e,f], Mustafa Soylak[e,g]

[a]Ankara University, Faculty of Pharmacy, Department of Analytical Chemistry, 06560, Ankara, Turkey
[b]Hacettepe University, Faculty of Sciences, Department of Chemistry, 06800, Ankara, Turkey
[c]Ankara University, Graduate School of Health Sciences, 06110, Ankara, Turkey
[d]Yozgat Bozok University, Science and Technology Application and Research Center, 66200, Yozgat, Turkey
[e]Erciyes University, Technology Research & Application Center (TAUM), 38039, Kayseri, Turkey
[f]Cankiri Karatekin University, Faculty of Science, Department of Chemistry, 18100, Cankiri, Turkey
[g]Turkish Academy of Sciences (TUBA), Çankaya, Ankara, Turkey

Corresponding authors: erk@pharmacy.ankara.edu.tr and gulbinkurtay@hacettepe.edu.tr

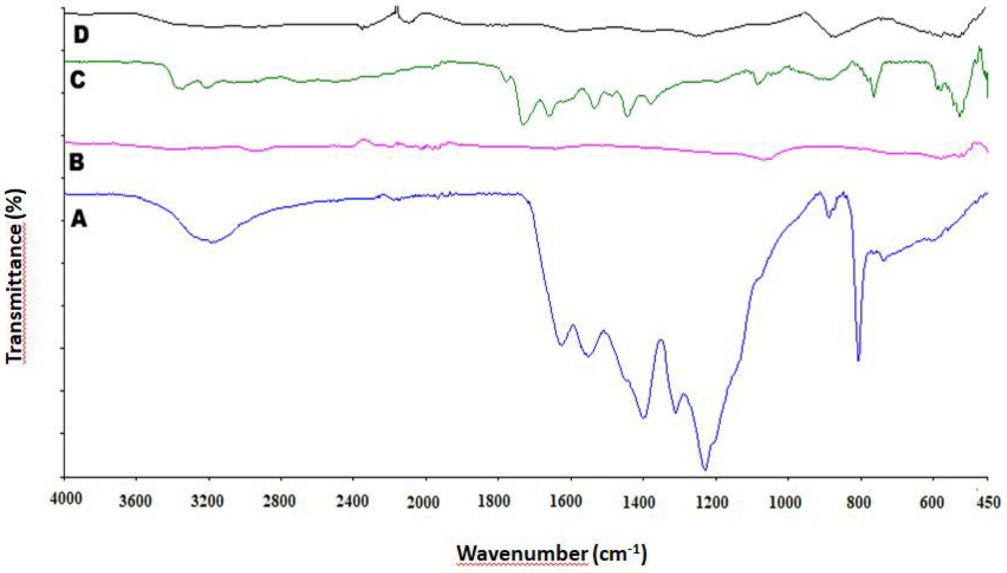

Figure S1. FT-IR spectrum of g-C_3N_4 (a), ND-COOH (b), g-C_3N_4@ND-COOH (c), and g-C_3N_4@ND-COOH@$MoSe_2$ (d) nanomaterials.

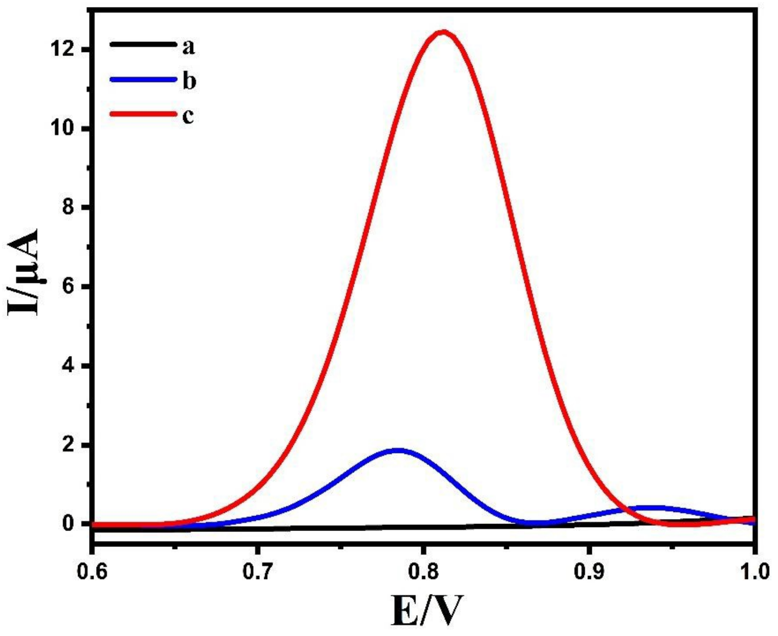

Figure S2. DPV results of 0.1 mM Mel in 0.1 M BR buffer at pH 2.0, blank (a), bare GCE (b) and g-C_3N_4@ND-COOH@$MoSe_2$/GCE (c).

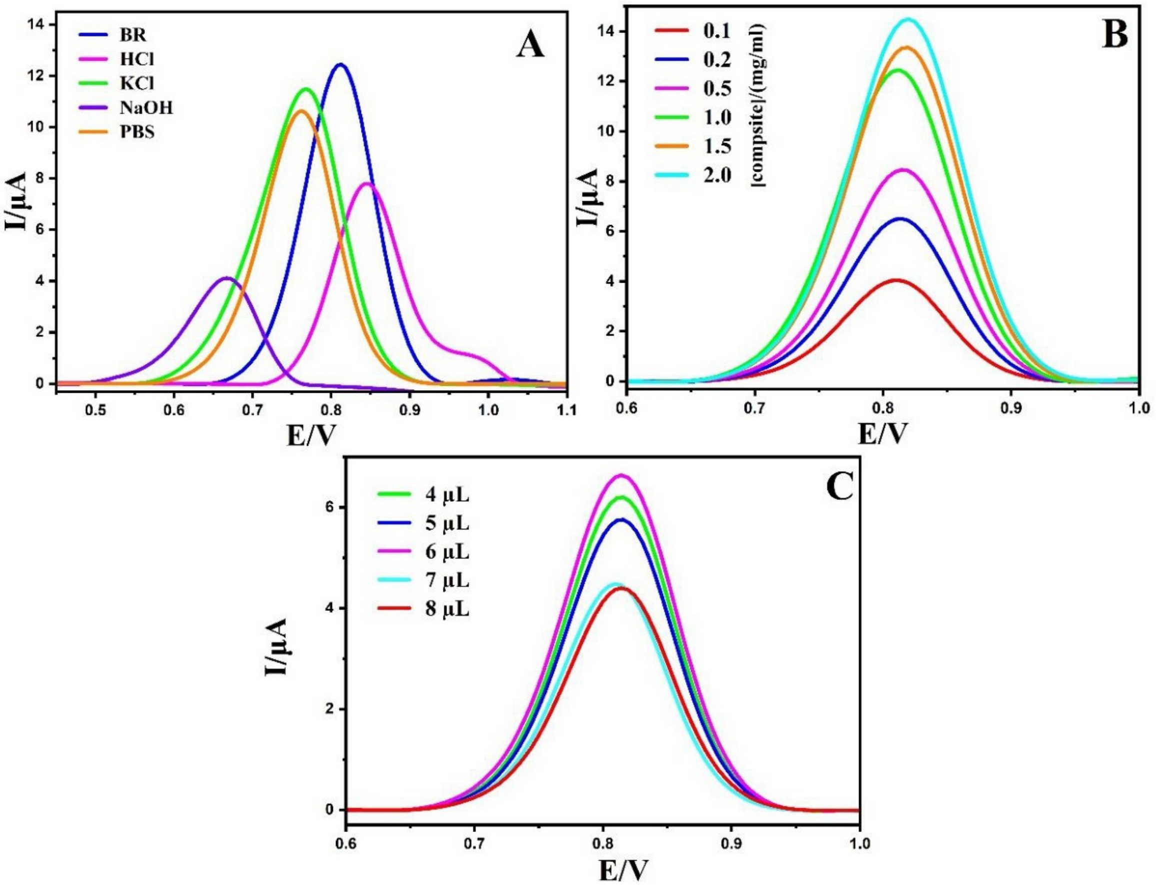

Figure S3. DPVs of different (A) electrolytes; (B) concentrations of the composite from 0.1 to 2.0 mg/mL; and (C) amounts of composite.

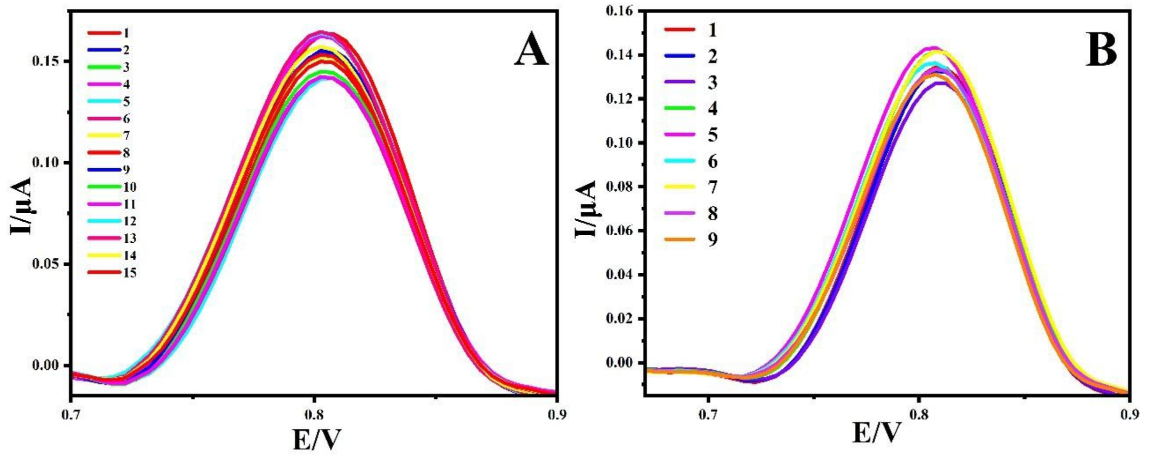

Figure S4. Repeatability (A) and reproducibility (B) of 0.1 mM MEL at g-C_3N_4@ND-COOH@$MoSe_2$/GCE in BR buffer at pH 2.
